# Interprofessional Management of (Risk of) Malnutrition and Sarcopenia: A Grounded Theory Study from the Perspective of Professionals

**DOI:** 10.2147/JMDH.S474090

**Published:** 2024-10-09

**Authors:** Sandra D Boxum, Sabien H van Exter, Jan-Jaap Reinders, Niek Koenders, Hans Drenth, Manon G A van den Berg, Michael Tieland, Sophie L W Spoorenberg, Evelyn J Finnema, Philip J van der Wees, Harriët Jager-Wittenaar

**Affiliations:** 1 Research Group Healthy Ageing, Allied Health Care and Nursing, Hanze University of Applied Sciences, Groningen, the Netherlands; 2 Science Department IQ Health, Radboud university medical center, Nijmegen, the Netherlands; 3 Department of Gastroenterology and Hepatology, Dietetics, Radboud university medical center, Nijmegen, 6500 HB, the Netherlands; 4 Center for Dentistry and Dental Hygiene, University Medical Center Groningen, University of Groningen, Groningen, the Netherlands; 5 Research Group Interprofessional Education (IPE), Lifelong Learning, Education and Assessment Research Network (LEARN), Research Institute SHARE, University Medical Center Groningen, University of Groningen, Groningen, the Netherlands; 6 Department of Rehabilitation, Radboud university medical center, Nijmegen, the Netherlands; 7 ZuidOostZorg, Organization for Elderly Care, Drachten, the Netherlands; 8 Department of Primary and Long-Term Care, University of Groningen, University Medical Center Groningen, Groningen, the Netherlands; 9 Center of Expertise Urban Vitality, Faculty of Sports and Nutrition, Amsterdam University of Applied Sciences, Amsterdam, the Netherlands; 10 School of Exercise and Nutrition Sciences, Institute for Physical Activity and Nutrition, Deakin University, Geelong, VIC, Australia; 11 Primary care group ‘Dokter Drenthe’, Assen, the Netherlands; 12 Health Science-Nursing Science and Education, University of Groningen, University Medical Center Groningen, Groningen, the Netherlands; 13 Research Group Living, Wellbeing and Care for Older People, NHL Stenden University of Applied Sciences, Leeuwarden, the Netherlands; 14 Research Group Nursing Diagnostics, Hanze University of Applied Sciences, Groningen, the Netherlands; 15 Department of Physiotherapy, Human Physiology and Anatomy, Faculty of Physical Education and Physiotherapy, Research Unit Experimental Anatomy, Vrije Universiteit Brussel, Brussels, Belgium

**Keywords:** malnutrition, sarcopenia, interprofessional, older adults

## Abstract

**Background:**

As our global population ages, malnutrition and sarcopenia are increasingly prevalent. Given the multifactorial nature of these conditions, effective management of (risk of) malnutrition and sarcopenia necessitates interprofessional collaboration (IPC). This study aimed to understand primary and social care professionals’ barriers, facilitators, preferences, and needs regarding interprofessional management of (risk of) malnutrition and sarcopenia in community-dwelling older adults.

**Methods:**

We conducted a qualitative, Straussian, grounded theory study. We collected data using online semi-structured focus group interviews. A grounded theory data analysis was performed using open, axial, and selective coding, followed by developing a conceptual model.

**Results:**

We conducted five online focus groups with 28 professionals from the primary and social care setting. We identified five selective codes: 1) Information exchange between professionals must be smooth, 2) Regular consultation on the tasks, responsibilities, and extent of IPC is needed; 3) Thorough involvement of older adults in IPC is preferred; 4) Coordination of interprofessional care around the older adult is needed; and 5) IPC must move beyond healthcare systems. Our conceptual model illustrates three interconnected dimensions in interprofessional collaboration: professionals, infrastructure, and older adults.

**Conclusion:**

Based on insights from professionals, interprofessional collaboration requires synergy between professionals, infrastructure, and older adults. Professionals need both infrastructure elements and the engagement of older adults for successful interprofessional collaboration.

## Introduction

As our global population continues to age, the prevalence of malnutrition and sarcopenia among older adults in the community is on the rise, significantly impacting quality of life and daily functioning.^1^,^2^ Malnutrition refers to a state that arises from a lack of intake or uptake of nutrition.^3^ Sarcopenia is a muscle disease that may result from physical inactivity, inflammation, and mitochondrial dysfunction but also from malnutrition.^4^ Both conditions are characterized by reduced muscle mass and in the case of sarcopenia, also loss of muscle strength. Moreover, both conditions contribute to an elevated risk of falls and fractures, leading to impaired daily activities, reduced independence, mobility issues, diminished quality of life, the necessity for admission to long-term care facilities, and premature increased mortality.^4^,^5^ A combined intervention consisting of nutrition and physical activity efficiently addresses malnutrition and sarcopenia by targeting nutritional deficiencies while simultaneously promoting muscle quality and strength synergistically.^6^,^7^ This combined strategy requires the collaboration of professionals from diverse disciplines, each contributing their unique expertise and skills.

Interprofessional collaboration (IPC) is essential for achieving optimal care outcomes.^8^ IPC involves different health or social care professions regularly coming together to provide services, characterized by shared accountability and interdependence between individuals, as well as clarity of roles and goals.^9^,^10^ This approach is more extensive than the more common multidisciplinary collaboration in healthcare, which often involves parallel efforts of professionals from different fields without integrated teamwork or shared goals.^11^

Numerous studies indicate that IPC in healthcare enhances effectiveness, leading to increased patient satisfaction, better-informed patients, better adherence to medication, lower readmission rates, and reduced complications related to illness.^12–15^ IPC also contributes to greater efficiency in the duration of treatment without altering the quality of care by decreasing the number of inpatient days, reducing (emergency) waiting times, facilitating quicker exchange of information, boosting productivity rates, and improving the accessibility, utilization, and flow of healthcare services.^16–20^ Cohesive actions, information, and expertise from various disciplines are necessary to address the complex needs of patients effectively.^21^

Despite the current evidence supporting the effectiveness of IPC and interventions that combine exercise and nutrition for addressing malnutrition and sarcopenia, the implementation of IPC in practice remains limited.^22^ Professionals encounter significant ideological, organizational, structural, and relational challenges when advocating for IPC.^23^ There is some understanding of the barriers to collaboratively addressing malnutrition, such as insufficient knowledge and awareness, limited access to dietetic services in primary care settings, and poor communication among professionals.^24–26^ However, information concerning IPC in the management of malnutrition and sarcopenia in primary care is still lacking. This underscores the current ambiguity of IPC in the context of the management of (risk of) malnutrition and sarcopenia among community-dwelling older adults.

Understanding professionals’ perspectives is essential for successful IPC in the management of (risk of) malnutrition and sarcopenia. Therefore, we evaluated the barriers and facilitators encountered by professionals as well as their preferences and needs regarding interprofessional treatment of malnutrition and sarcopenia in community-dwelling older adults.

## Methods

### Study Design

We conducted a qualitative study through focus groups using grounded theory methods as delineated by Strauss and Corbin.^27^ This Straussian approach follows a systematic, interpretative process to produce a conceptual model through an iterative data analysis grounded in the perspectives and experiences of professionals working in the primary or social care setting, further indicated as “professionals”. To ensure transparent and rigorous reporting of the study’s findings, the researchers adhered to the 32-item checklist of the Consolidated Criteria for Reporting Qualitative Studies (COREQ).^28^

### Eligibility Criteria and Recruitment

Professionals working in primary or social care who encountered (risk of) malnutrition and/or sarcopenia among community-dwelling older adults were eligible to participate. Proficiency in speaking, reading, and writing Dutch was required. We employed a purposive maximum variation sampling strategy to aim for diversity in profession and years of work experience, capturing a wide range of perspectives within each focus group. Researchers disseminated a call for participation through professional networks, and eligible participants expressed interest by contacting the researchers via Email or telephone. We aimed for participants from various regions in the Netherlands, including the rural provinces of Drenthe and Friesland and the cities of Nijmegen and Amsterdam, enabling the exploration of diverse collaboration contexts and practices. Informed consent, including permission to publish anonymized quotes and participant characteristics, was obtained from participants prior to each focus group session. No prior relationship was established with participants before the study commenced.

### Data Collection

Data were collected using online focus group sessions via Microsoft Teams (version range 1.6.00.6754–1.6.00.27573, Microsoft). Each focus group lasted two hours, including a 10-minute introduction and a 5-minute mid-session break. The focus groups comprised five to seven participants to maintain manageable online discussions. One researcher (SB) moderated the focus group sessions using an interview guide that was adapted between focus groups (Appendix A and B), a second researcher co-moderated (JJR), while a third researcher was taking notes as documenter (SvE). Following each session, SB, JJR, and SvE discussed field notes and made necessary adjustments to the interview guide. The topics in the interview guide were derived from the Meta-Model of Interprofessional Development.^29^ Questions comprised topics such as professionals’ experiences with malnutrition and sarcopenia in older adults, their current collaboration practices, facilitators and barriers for IPC, and their vision of the ideal IPC. The questions also delved into the role of older adults in IPC. In addition to the interview questions, the concept of IPC was clarified after a short discussion on the concept among professionals to ensure a shared understanding of its definition. Focus groups were video recorded and transcribed verbatim by a third party (Marinka^®^ Transcriberen). Data collection ended after reaching theoretical saturation, at which point no new selective codes emerged.

### Reflexivity

The team’s diverse expertise enriched the exploration of interprofessional management of malnutrition and sarcopenia. SB, a physiotherapist, offered insights from primary healthcare practice, while SvE, with a background in nutrition science, focused on considerations regarding nutrition assessment and treatment. JJR, a work and organizational psychologist, contributed insights into the dynamics influencing IPC. SB and SvE were PhD candidates, and JJR held a postdoctoral position. SB had experience conducting interviews and was trained in qualitative research and focus group moderation. In addition to SB, JJR, and SvE, the research team involved in the analysis consisted of individuals with diverse backgrounds, including nutrition and physical activity [MT], dietetics [MvdB, HJW], physiotherapy [NK, HD, PvdW], gerontology [HD], and qualitative research [NK], providing a comprehensive perspective on the analysis. All researchers work in the fields of malnutrition, sarcopenia, and/or IPC and, therefore, may have preconceived notions about the importance of these concepts. Efforts were made to mitigate potential biases by promoting open discussions and critical reflections within the research team.

### Data Analysis

Grounded theory data analysis followed three steps: open coding, axial coding, and selective coding.^27^ SB and SvE independently read, re-read, and coded the transcripts using open codes. Subsequently, they compared their codes, engaging in open and constructive dialogue until a consensus was reached. A constant comparative approach was employed to systematically compare participant responses across focus groups. Subsequently, axial codes were produced after analyzing the focus group sessions. After all focus groups had finished, SB and SvE created selective codes to organize the axial codes and identify connections in the data. Following the completion of the three stages of analysis by SB and SvE, all codes were reviewed within the research team to enhance mutual understanding and achieve consensus through five meetings with the research team. The first session focused on enhancing the open and axial codes. The second and third sessions were dedicated to discussing the axial and selective codes. The fourth and fifth sessions were dedicated to constructing the conceptual model.

### Trustworthiness

Rigorous methodological practices contribute to enhancing the trustworthiness of the research findings. ATLAS.ti software (version 8.4, Scientific Software Development GmbH) facilitated data aggregation and analysis, promoting transparency and authenticity in code descriptions through participant quotes. Independent readings and coding enhanced thoroughness and addressed potential individual blind spots, strengthening the credibility of the interpretations. Furthermore, the constant comparison approach further bolstered credibility by systematically comparing data points and codes throughout the analysis process. This method ensured that interpretations were grounded in the data and allowed for the refinement of axial codes. The ongoing refinement of the interview guide, guided by insights from discussions between the focus group sessions, increased the depth of research findings by validating data collection methods and exemplified researchers’ commitment to critical reflection on their biases and assumptions throughout the research process. Analytical memos were documented throughout the data analysis process to record decisions and interpretations. This enabled the research team to confirm the study’s findings independently of the first authors’ biases or influences, further enhancing the confirmability of the findings.

## Results

Five online focus groups were conducted, involving a total of 28 participants representing four distinct regions across the Netherlands. Focus group sessions lasted 105 minutes on average (range 98–113 minutes). Theoretical saturation was achieved after the fifth focus group session. Although most contacted professionals initially expressed their willingness to participate, one individual who initially agreed failed to attend the session and did not respond to subsequent follow-up emails. Additionally, another professional was unable to attend on the agreed date due to scheduling conflicts, while a third had to cancel due to illness. Participant characteristics are detailed in Table 1.

**Table 1:** Participant Characteristics

	Participants (n)
**Total Participants**	28
**Sex**	
Female	24
Male	4
**Profession**	
Dementia case manager	5
Dietitian	5
District nurse	5
General practitioner (GP)	2
GP practice assistant	4
(Geriatric) physiotherapist	5
Nurse practitioner	1
Social worker	1
**Participants per region**	
Friesland	5
Drenthe	13
Nijmegen	5
Amsterdam	5
**Years of working experience in current expertise**	
0–9	14
10–19	9
20–29	1
30–39	4

In our analysis, we identified five selective codes encapsulating the barriers, facilitators, preferences, and needs of professionals regarding IPC in managing (risk of) malnutrition and sarcopenia among community-dwelling older adults. The following sections will explore these selective codes and their associated axial codes in depth. Table 2 lists an overview of the selective and axial codes.

**Table 2:** Selective Codes with Axial Codes

**Selective code 1: Information exchange between professionals must be smooth**
1A: Professionals experience non-interoperable EHRs as a barrier to IPC
1B: Professionals prefer to work in one shared EHR
1C: Professionals need different communication tools
1D: Professionals prefer to know other professionals in person
1E: Professionals prefer to have other professionals nearby
1F: Professionals need knowledge about malnutrition and sarcopenia to ensure smooth communication
**Selective code 2: Regular consultation on the tasks, responsibilities, and extent of IPC is needed**
2A: Professionals need structural meetings
2B: Professionals need agreements regarding tasks and responsibilities
2C: Professionals face the barrier that not all involved professionals can attend structural meetings
2D: Professionals prefer a balance in the content and extent of information exchange
2E: Professionals experience time constraints as a barrier to IPC
2F: Professionals think receiving financial compensation would facilitate regular interprofessional consultations
**Selective code 3: Thorough involvement of older adults in IPC is preferred**
3A: Professionals prefer that older adults can be self-determined in their care
3B: Professionals experience difficulties in engaging older adults as a barrier to IPC
3C: Professionals prefer shared decision-making with older adults regarding healthcare choices
**Selective code 4: Coordination of interprofessional care around the older adult is needed**
4A: Professionals need a coordinator in the team
4B: Professionals need the timely involvement of relevant other professionals in the healthcare process
4C: Professionals view the reluctance of older adults to engage other healthcare professionals as a barrier
**Selective code 5: IPC must move beyond healthcare systems**
5A: Professionals need organizations outside the healthcare sector to support IPC
5B: Professionals need informal caregivers and family to be involved in IPC
5C: Professionals think financial compensation from insurance to the older adult would facilitate IPC

**Notes**: “Professionals” are professionals who work in primary or social care.

**Abbreviations**: EHR, Electronic health record; IPC, Interprofessional collaboration.

### Selective Code 1: Information Exchange Between Professionals Must Be Smooth

Professionals seek an information exchange process that is seamless, uninterrupted, and free from obstacles, all with the overarching goal of enhancing IPC.

#### Axial Code 1A: Professionals Experience Non-Interoperable EHRs as a Barrier to IPC

Professionals explained that the current non-interoperability of electronic health records (EHRs) complicates information exchange. Dietitians and physiotherapists emphasized the difficulties arising from using different systems that do not communicate with other EHRs, resulting in duplicated efforts and challenges in transitioning between platforms. However, it was recognized that interoperability between different EHRs could improve collaboration.

District nurse: One of the biggest issues for me in collaboration is that the systems don’t quite match up. We use an electronic health record, but I can’t integrate the files of another electronic health record into it. So, I end up having to fill out a separate care plan, which is a bit of a hassle.

#### Axial Code 1B: Professionals Prefer to Work in One Shared EHR

Professionals would prefer a shared EHR to facilitate shared care plans and goals. However, the lack of uniformity in EHRs among healthcare institutions was viewed as a barrier to effective communication. Despite challenges, professionals maintained a positive perspective on the concept of a shared EHR, listing advantages such as accessible contact, efficient communication, and time efficiency. Additionally, enabling patients to log into their health records was perceived as a positive development, allowing them access to information about their health.

Dietitian: It becomes more challenging when you don’t work with a shared system; coordinating requires speaking and exchanging secure emails. You may reach one person, but not necessarily colleagues. With a shared electronic health record, everyone can access it, including caregivers or partners. I find that very convenient.

#### Axial Code 1C: Professionals Need Different Communication Tools

Professionals seek to balance traditional and modern communication methods to ensure effective and efficient communication. Professionals pointed out various communication barriers, such as different work schedules, inconvenient phone calls, and challenges in scheduling face-to-face and online meetings. When it comes to exchanging critical information, professionals still prefer telephone contact, considering it faster than sharing comprehensive reports. Nevertheless, professionals think sharing comprehensive EHRs is beneficial for future or further referencing of information. Additionally, professionals acknowledged the benefits of using digital communication tools, including online interactions and the use of apps for secure short messaging between professionals.

Dementia case manager: I have noticed that the most effective way to communicate is through quick calls and emails. Occasionally, if it’s not related to medical matters, you can shoot a message or drop by to schedule a meeting. I have found that works well, just a brief phone call.

#### Axial Code 1D: Professionals Prefer to Know Other Professionals in Person

Professionals highlighted the importance of personal connections with other professionals, emphasizing their positive impact on building sustainable collaborations. However, they identified staff turnover as a significant barrier, and the collaboration with various healthcare institutions complicated the process of getting to know others well.

General practitioner: It’s about really knowing each other. Having those face-to-face interactions makes it easy to give a call, shoot a message, or even drop by. That personal connection is important to me. If I’ve spoken to someone even just once, it breaks down barriers.

#### Axial Code 1E: Professionals Prefer to Have Other Professionals Nearby

Professionals preferred in-person, face-to-face interactions, citing it as facilitative for IPC and efficient communication. Working in the same building or sharing an office space was experienced as a practical way to establish direct lines of communication. Professionals already working with various disciplines within a single organization reported experiencing the benefits of these direct lines of communication, enabling quick and efficient interaction.

GP practice assistant: I prefer to keep it informal and choose professionals who are close to me. So, those in the same building or with whom I collaborate well. People I can rely on, and I know that if there’s anything, they’ll give me a call.

#### Axial Code 1F: Professionals Need Knowledge About Malnutrition and Sarcopenia to Ensure Smooth Communication

Professionals noted differences in knowledge levels concerning malnutrition and sarcopenia. Dementia case managers, district nurses, and practice assistants acknowledge a knowledge deficit in these areas despite some reported specific training on this topic. Nonetheless, professionals expressed a need for further education in malnutrition and sarcopenia. The lack of understanding often challenges comprehending each other’s reports. They stressed that improved knowledge could aid in the early detection of malnutrition and sarcopenia. Dietitians and physiotherapists were recognized for their expertise in malnutrition and sarcopenia and were identified as valuable resources for disseminating this knowledge.

### Selective Code 2: Regular Consultation on the Tasks, Responsibilities, and Extent of IPC is Needed

Professionals acknowledged the importance of structural meetings for fostering effective communication and collaboration.

#### Axial Code 2A: Professionals Need Structural Meetings

Beyond their functional aspects, professionals acknowledged online or in-person team meetings as valuable networking opportunities, enhancing professional relationships and connectivity. Preferences for meeting frequency varied, with suggestions ranging from monthly to every eight weeks, emphasizing productivity when multiple disciplines can attend. Furthermore, there was a preference for increased involvement of dietitians, physiotherapists, and social care workers in team meetings because they are currently not always involved. Structural meetings were viewed as valuable opportunities for mutual learning and ensuring alignment among team members.

General practitioner: Frequently, this [structural meetings] adds a valuable boost to the treatment, as everyone is once again on the same page with a particular client. Moreover, it’s often an opportunity to gather insights into someone’s working style, thought processes, and, oh, perhaps I should give him a ring sometime.

#### Axial Code 2B: Professionals Need Agreements Regarding Tasks and Responsibilities

Professionals underscored the significance of enhanced coordination and well-defined practical agreements regarding tasks and responsibilities. This need extends to establishing clear and effective protocols among professionals, ensuring precise work arrangements, and optimizing task distribution. Additionally, a preference was mentioned to streamline task allocation and coordinate meeting attendance for optimal collaboration.

Dementia case manager: In one situation, I came into the picture, and the general practitioner had already brought in a dietitian. If you establish a connection with the dietitian like, ‘Well, I’m involved now too,’ maybe you should set clear agreements right away.

#### Axial Code 2C: Professionals Face the Barrier That Not All Involved Professionals Can Attend Structural Meetings

Professionals believed structural meetings were most productive when multiple professionals from diverse disciplines were involved. Low commitment due to heavy workloads and staff turnover were mentioned as barriers to attending those meetings. Planning meetings was seen as a complex task that demands seamless coordination among professionals from multiple organizations. It remained unclear who should undertake this responsibility, as multiple professionals would be capable of this task. Physiotherapists and dietitians were often absent at these structural meetings, suggesting that attendance could be more feasible if they received specific invitations tailored to meetings involving cases where their expertise is directly relevant.

#### Axial Code 2D: Professionals Prefer a Balance in the Content and Extent of Information Exchange

Professionals emphasized that not every older adult with (risk of) malnutrition or sarcopenia should be discussed at every meeting. Focused discussions are preferred, only discussing those patients that require attention at that moment. Attention was also given to selectively sharing information between professionals by managing the flow of information. Sharing substantial amounts of information can lead to a flood of data, which means professionals cannot keep track of everything. Finding the appropriate equilibrium was considered crucial, as excessive sharing can overwhelm and hinder efficiency, while too little information leaves professionals uninformed about each other’s treatment plans. Professionals prefer to develop a shared care plan, especially in complex cases. Additionally, professionals stressed the importance of considering various aspects of information exchange, including addressing critical health signals, making referrals to other professions, managing noncompliance, marking the start and end of treatment, and identifying instances where someone requires additional or specialized care.

#### Axial Code 2E: Professionals Experience Time Constraints as a Barrier to IPC

Time constraints emerged as a significant barrier to IPC. High administrative burdens and busy schedules contribute to a shortage of time among professionals. IPC was perceived as potentially time-consuming, particularly when engaging older adults. However, professionals expressed a positive perspective on the efficiency of virtual collaboration, finding online discussions to be both time-saving and cost-saving.

#### Axial Code 2F: Professionals Think Receiving Financial Compensation Would Facilitate Regular Interprofessional Consultations

Professionals emphasized that receiving financial compensation would facilitate IPC. They noted disparities among disciplines regarding compensation currently exist, with physiotherapists, dietitians, general practitioners, and practice assistants often facing a lack of financial remuneration compared to other disciplines, such as dementia case managers, district nurses, and social care workers. Professionals identified diverse funding streams and the absence of financial compensation as significant barriers to effective IPC.

### Selective Code 3: Thorough Involvement of Older Adults in IPC is Preferred

Professionals advocated for actively involving older adults and preserving their self-determination while recognizing the complexities and challenges that may impede this goal.

#### Axial Code 3A: Professionals Prefer That Older Adults Can Be Self-Determined in Their Care

Professionals expressed a strong need for older adults to be self-determined and to be in charge of their healthcare decisions. Professionals acknowledged that preserving self-determination comes with difficulties due to factors like stress, cognitive issues, and mental challenges. Additionally, professionals stated safety is always the number one priority and may take precedence over self-determination in some instances.

#### Axial Code 3B: Professionals Experience Difficulties in Engaging Older Adults as a Barrier to IPC

Professionals faced challenges in actively involving older adults, presenting a significant barrier to IPC. These challenges parallel the difficulties encountered in maintaining self-determination for older adults with dementia. Additionally, professionals mentioned factors such as the lack of accountability among older adults for their health, avoidance of seeking care, low comprehension, low health literacy, and a lack of motivation can further hinder engagement. Finally, professionals noted that engaging older adults is possible when sufficient time is available; however, this resource is often scarce.

District nurse: I believe one’s understanding, low literacy, and willingness are all factors [in engaging the older adults] when I look at my neighborhood.

#### Axial Code 3C: Professionals Prefer Shared Decision-Making with Older Adults Regarding Healthcare Choices

Professionals emphasized the importance of ensuring that the need for care arises from, or aligns with, the older adults themselves rather than being solely influenced by their families. They advocate for engaging in conversations directly with older adults instead of discussing them among professionals or family, believing this approach would be appreciated by the older adults and improve treatment. Professionals stated that while shared decision-making is already a frequent practice in many disciplines, communication is essential to facilitate this collaborative decision-making process. A shared decision could also mean a person does not want to continue treatment or may not want to engage in shared decision-making, even if a professional would consider shared decision-making preferable.

General practitioner: We consider what suits each person and how they view it. If someone doesn’t want to participate, you can encourage or revisit the idea occasionally, but if they’re not willing, that’s where it ends. It’s about making an individual plan, keeping in mind what’s feasible and the person’s preferences. We may have our opinions, but if the patient doesn’t see it as an issue, we revisit it at a later time.

### Selective Code 4: Coordination of Interprofessional Care Around the Older Adult is Needed

Professionals highlighted the importance of a designated coordinator to ensure clarity and timely involvement of other relevant professionals.

#### Axial Code 4A: Professionals Need a Coordinator in the Team

Professionals emphasized the need for a coordinator in the team. They noted that the involvement of multiple professionals in caring for an older adult often results in a lack of clarity for the individual receiving care. According to professionals, the absence of coordinated care can lead to an open-ended care process. Professionals believe that older adults value coordination, especially when they struggle to maintain an overview of their healthcare. Professionals, such as a general practitioner, dementia case manager, or district nurse, could potentially assume this role. Professionals also suggested that an informal caregiver could fulfill this coordinating function.

#### Axial Code 4B: Professionals Need the Timely Involvement of Relevant Other Professionals in the Healthcare Process

The team of professionals needs to be expanded promptly to include relevant professionals for treating (risk of) malnutrition and sarcopenia. Physiotherapists and dietitians expressed concerns about the delayed recognition of their potential value, often attributed to a lack of knowledge among other professionals and older adults. This delayed involvement of professionals is evident from the initial contact and the preliminary stages of malnutrition or sarcopenia, extending to potential relapses after treatment.

Dietitian: Yesterday, I received a referral from a general practitioner stating that someone lost ten kilograms in the past year. The general practitioner had provided some nutritional drink bottles, but we weren’t involved. It felt like things went wrong from the start. […] Sometimes we are involved too late.

#### Axial Code 4C: Professionals View the Reluctance of Older Adults to Engage Other Healthcare Professionals as a Barrier

Professionals perceive a lack of awareness among older adults regarding the added value and roles provided by various disciplines. This was particularly seen by physiotherapists, dietitians, and dementia case managers. Notably, professionals emphasized that dietitians face challenges due to an unfavorable image among older adults. Furthermore, professionals explained that older adults are motivated to maintain independence for as long as possible. This desire for independence may lead older adults to resist involvement from other professionals, posing a barrier to IPC.

GP practice assistant: I encounter individuals with resistance, especially the 88-year-olds who are like, ‘I’ve always managed on my own, so I don’t want any help.’ They don’t allow themselves the luxury of a physiotherapist. It’s not that they refuse to exercise; it’s more like, ‘I’ve always handled things on my own.

### Selective Code 5: IPC Must Move Beyond Healthcare Systems

Professionals emphasize the need for support from organizations outside the healthcare sector to enhance and facilitate collaboration.

#### Axial Code 5A: Professionals Need Organizations Outside the Healthcare Sector to Support IPC

Professionals emphasized the importance of support from organizations outside the healthcare sector to facilitate IPC. Social care workers were highlighted as valuable allies, particularly in addressing lifestyle improvements and social aspects. They have the time and expertise to gain the trust of older adults. It was mentioned that this trust and sense of rapport could facilitate the acceptance of other professionals relevant to managing malnutrition and sarcopenia. Additionally, daycare and domestic assistance could play a role in encouraging physical activity and promoting healthy eating habits among older adults, according to professionals. However, professionals pointed out that daycare facilities used to have the older adults spend their day there cooking a nutritious meal together for the group. Professionals have expressed that daycare facilities used to involve older adults living at home cooking nutritious meals together as a group. However, government regulations and financial constraints hinder communal cooking in daycare facilities. This results in the provision of pre-cooked and less nutritious meals, which may be less optimal for older people at risk for malnutrition and sarcopenia. Professionals expressed the preference to engage the local government in initiatives focused on preventing malnutrition and sarcopenia. This includes providing information and creating opportunities for older adults to develop and maintain a healthy lifestyle.

#### Axial Code 5B: Professionals Need Informal Caregivers and Family to Be Involved in IPC

Professionals emphasized the vital role of involving informal caregivers and families in IPC. They stressed the importance of discussing the wishes and goals of the older adult and their family members. Informal caregivers and family members were recognized for their essential role in motivating the older adult, particularly during and after treatment, especially in cases where dementia is involved.

#### Axial Code 5C: Professionals Think Financial Compensation from Insurance to the Older Adult Would Facilitate IPC

Professionals advocated financial compensation through healthcare insurance in facilitating the interprofessional management of older adults at risk of malnutrition or sarcopenia. Professionals expressed disappointment that financial factors often significantly influence older adults’ healthcare decisions. Some individuals feel compelled to prioritize financial considerations when making healthcare choices, and in some instances, older adults decline care due to financial constraints. The discontinuation of interventions due to limited or no reimbursement from healthcare insurers was acknowledged as a substantial barrier. Insufficient financial support poses challenges for both older adults and healthcare providers, affecting the accessibility of essential care. The lack of adequate financial support was especially highlighted concerning treatment by a dietitian or physiotherapist.

Dementia case manager: There are sometimes financial considerations at play, preventing patients from seeking help. They might not mention it initially, but through further inquiry, you may discover that. For instance, someone might not have supplementary insurance and want to know the cost beforehand. That can also be a hindering factor for effective collaboration.

### Conceptual Model

**Figure 1:**
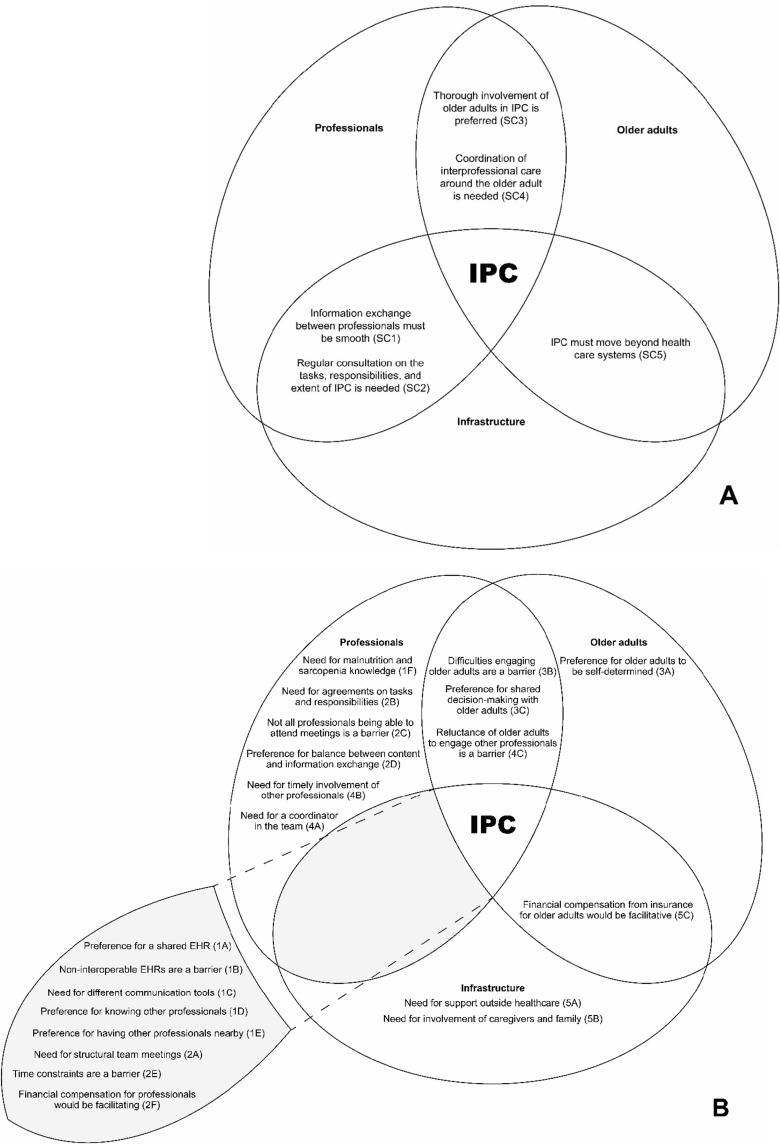
Conceptual model of interprofessional collaboration in the management of (risk of) malnutrition and sarcopenia: A professional’s perspective. (**A**) Interconnectedness of selective codes among the dimensions of professionals, infrastructure, and older adults. (**B**) Interconnectedness of axial codes among the dimensions of professionals, infrastructure, and older adults.

The conceptual model was derived from the findings of this study. Selective codes (depicted in Figure 1, Section A) and underlying axial codes (shown in Figure 1, Section B) were categorized under three main dimensions: professionals, infrastructure, and older adults, represented by ellipses. Overlapping areas illustrate interdependencies and interconnectedness among these dimensions. The term “professionals” refers to professionals from various disciplines in health and social care. The term “infrastructure” encompasses diverse factors and organizations that influence IPC, including technical and social aspects such as time and financial resources. The term “older adults” represents the patients or clients undergoing management of (risk of) malnutrition and/or sarcopenia.

## Discussion

In this qualitative study, we aimed to understand the barriers, facilitators, preferences, and needs encountered by professionals regarding the interprofessional management of (risk of) malnutrition and sarcopenia in community-dwelling older adults. The insights gained from professionals indicated that achieving IPC requires synergy among professionals, infrastructure, and older adults.

Within the dimension of professionals, the presence of a coordinator, clear agreements on tasks and responsibilities, and sufficient knowledge of malnutrition and sarcopenia are needed, according to professionals. Additionally, professionals prefer a balance in the exchange of information but face barriers such as the inability of all professionals to attend structural meetings. Regarding the interconnection between the dimensions of professionals and infrastructure, professionals addressed non-interoperable EHRs and time constraints as barriers to IPC. They expressed a preference for a shared EHR system, emphasized the value of in-person connections, and preferred to have other professionals nearby. Financial compensation for professionals was perceived as a facilitating factor for IPC. Professionals also recognized the value of both traditional and modern communication tools and stressed the need for structural meetings to facilitate collaboration. In the dimension of infrastructure, professionals underscored the need to involve informal caregivers, families, and external organizations such as insurance companies to facilitate IPC. Regarding the interconnection between infrastructure and older adults, professionals recognized financial compensation from insurance to older adults as facilitative for IPC. Finally, professionals expressed a preference for older adults to be self-determined and to be in charge of their healthcare decisions.

Our study aligns with previously established findings on IPC in a primary care setting. Facilitators mentioned in previous research, such as organizing regular meetings, employing clear communication routines or information channels, working at the same location, and establishing relationships, were corroborated by our findings.^30^,^31^ Professionals in our study further underscored these facilitators by emphasizing the positive impact of knowing each other in person, having other professionals nearby, regular acquaintance with other professionals, using facilitative communication tools, and the importance of clear protocols. Research has shown that regular interpersonal contact among team members influences the sense of belonging of professionals, positively impacting their intentions to exchange knowledge.^32^,^33^ Therefore, fostering an environment that facilitates regular interaction and clear communication can contribute not only to effective IPC but also to the willingness of team members to share valuable knowledge.

Our findings validate time constraints as a barrier to effective IPC in primary care, as described in the literature.^20^,^34^ Professionals in our study echoed these barriers, particularly regarding structural team meeting attendance. Acknowledging that efficient communication positively impacts available time, addressing communication processes becomes crucial to alleviate time-related challenges. Strategies such as flexible scheduling or incorporating virtual meeting options may prove essential. Flexible scheduling allows professionals to join and leave meetings when needed. Using flexible scheduling for face-to-face and virtual meetings would offer solutions to strengthen team dynamics and overcome time-related challenges while preserving the benefits of regular face-to-face interactions. Furthermore, professionals emphasized the need for enhanced coordination and clear agreements regarding tasks and responsibilities. Clarity of goals, team roles, and responsibilities is not only essential for effective IPC but is also consistently emphasized in the definitions of interprofessional work across various clinical settings and in different national contexts.^35^

Moreover, according to our findings, financial compensation for the professional and the older adult with malnutrition and/or sarcopenia can further support IPC. Professionals may find it challenging to collaborate during their own time, which is now often necessary. Older adults with malnutrition or sarcopenia frequently face inadequate compensation from health insurers for services provided by physiotherapists or dietitians, leading to reluctance to involve these professionals in their care team. These observations are in line with the literature identifying financial support and supportive policies as facilitators of IPC.^31^,^36^ The current health and social care payment models are often fragmented or based on fee-for-service systems.^37^,^38^ Such models can create conflicting financial incentives that discourage collaboration between professionals and limit comprehensive care. Integrated payment models, which streamline payments across providers and encourage shared responsibility, are suggested as promising solutions to enhance care coordination and improve patient outcomes.^39^,^40^

Professionals’ preference for shared decision-making with older adults aligns with the interprofessional communication competency outlined by the Interprofessional Education Collaborative (IPEC).^41^ This competency emphasizes adapting communication strategies to accommodate diverse patient populations, including those facing cognitive impairments or limited health literacy. Acknowledging and addressing these challenges in patient communication aligns with the overarching goal of providing patient-centered care, ensuring that communication methods are tailored to older adults’ unique needs and circumstances.

While our study primarily highlighted organizational and structural barriers to IPC, a recent study on primary healthcare centers in Qatar also identified cultural barriers, including “fear culture” and professional hierarchies.^36^ Concerns about adverse consequences when reporting errors were noted to discourage open communication among professionals. These findings reflect differences in organizational structures and cultural norms between the two contexts. Qatar’s healthcare system may have more pronounced hierarchical structures, which could contribute to these cultural barriers. In contrast, the context of our study may have involved fewer cultural differences and hierarchical structures, which explains why cultural barriers were not prominent in our findings. It is important to remain mindful that barriers to IPC can vary significantly across different healthcare systems and cultural environments.

### Strengths and Limitations

We employed a rigorous grounded theory methodology to strengthen our research. Two independent researchers coded the data, ensuring the reliability of our findings and minimizing interpretation bias.

Another strength of our study is the inclusion of professionals from diverse disciplines and regions, with a sample demographic that accurately reflects real-world settings. A significant proportion of female participants and individuals with 0–9 years of work experience in their current expertise were included in our study. This demographic profile closely mirrors the typical workforce in the care for older adults, which is predominantly female on a global scale.^42^ For instance, in the Netherlands, approximately 80% of those employed in health and social care are female.^43^ Several factors contribute to the prevalence of individuals with less experience in their current roles, including frequent role transitions due to additional training and a notable representation of younger professionals in the health and social care sectors.^44^

Furthermore, the strategic time gaps between focus groups allowed for the iterative refinement of questions, facilitating a nuanced exploration of axial and selective codes. The focus group format’s efficiency, enriched with symbolic interactionism principles, encouraged lively discussions and idea generation. These exchanges enhanced the depth and richness of discussions by making participants attribute meaning and interpretations to shared ideas, words, and interactions.

Nevertheless, we recognize certain limitations inherent to our study. Firstly, although focus groups are a proper method for this grounded theory research, they could have been expanded to include more types of methods, such as individual interviews, team observations or participant diaries. These could have further enriched the data. Secondly, all participating professionals may have had a baseline interest in IPC as they voluntarily participated in a focus group on this subject. We cannot rule out the possibility of missing results and insights from professionals less interested in IPC through selective sampling. This might explain why only one social care worker participated, as they may not have been drawn to the initial call for participation even though an invitation for study participation was specifically given to welfare organizations. The abundance of participating healthcare professionals naturally caused focus groups to shift towards a health-focused viewpoint. However, including participants with a strong motivation for IPC is likely to have contributed to the generation of rich data.^45^ Thirdly, despite our efforts to maintain objectivity and engage in self-reflection, we acknowledge that researchers inevitably brought their perspectives and biases to this qualitative study. To address this, we employed researcher triangulation during the analysis, involving a diverse team to enhance the study’s robustness and minimize the impact of individual prejudices and assumptions throughout the research process.

#### Implications for Clinical Practice and Future Research

The conceptual model emphasizes the importance of synergy among multiple dimensions to achieve successful IPC in managing malnutrition and sarcopenia. To achieve this, an interprofessional team should establish explicit protocols and agreements to ensure effective communication and information exchange. Improving infrastructure, such as implementing interoperable or shared EHRs and regular team meetings, is essential for facilitating the exchange of knowledge and information. Additionally, involving social workers, informal caregivers, and local governments can provide valuable support to the interprofessional team and older adults. Furthermore, older adults must be involved in their healthcare decisions despite challenges such as cognitive issues and low health literacy. Professionals can encourage this involvement by inviting patients and their support networks to participate actively in the decision-making process, considering their individual needs and circumstances.

Moving forward, research efforts should prioritize understanding and enhancing the practical implementation of IPC to address (risk of) malnutrition and sarcopenia among community-dwelling older adults. Gathering insights into the wishes and needs of older adults is essential to ensure that IPC truly centers around the patient’s perspective. Additionally, assessing the feasibility and effectiveness of implementing IPC in real-world settings is also imperative. One potential approach to achieve these goals is by first co-designing an interprofessional care pathway tailored to address the specific needs of older adults with malnutrition and sarcopenia, and professionals needed for IPC. Subsequently, implementing and evaluating its impact. Such research pursuits can offer valuable insights into enhancing the quality of care, improving job satisfaction among healthcare professionals, managing healthcare costs, and improving the health outcomes of older adults with (risk of) malnutrition and sarcopenia.

## Conclusion

Based on insights from professionals, interprofessional collaboration requires synergy between professionals, infrastructure, and older adults. Professionals need both infrastructure elements and the engagement of older adults for successful interprofessional collaboration. Regarding the interconnection between the professionals’ and infrastructure dimensions, professionals addressed non-interoperable electronic health records and time constraints as barriers to interprofessional collaboration. Professionals expressed a preference for a shared electronic health record, emphasized the value of in-person connections, and preferred to have other professionals nearby. Financial compensation for professionals was perceived as a facilitator for interprofessional collaboration. Professionals also recognized the value of both traditional and modern communication tools and stressed the need for structural meetings. Concerning the interconnection between infrastructure and older adults, professionals noted that financial compensation from insurance for older adults would facilitate interprofessional collaboration.

This study contributes to a deeper understanding of interprofessional collaboration in primary care settings and offers valuable insights for professionals, researchers, and policymakers.
